# Mutagenesis and Adaptation of the Psychrotrophic Fungus *Chrysosporium pannorum* A-1 as a Method for Improving β-pinene Bioconversion

**DOI:** 10.3390/molecules25112589

**Published:** 2020-06-02

**Authors:** Mateusz Kutyła, Jan Fiedurek, Anna Gromada, Krzysztof Jędrzejewski, Mariusz Trytek

**Affiliations:** Department of Industrial and Environmental Microbiology, Faculty of Biology and Biotechnology, Maria Curie-Skłodowska University, Akademicka 19, 20-033 Lublin, Poland; mateusz.kutyla@umcs.pl (M.K.); janek@umcs.pl (J.F.); anna.gromada@umcs.pl (A.G.); krzysztof.jedrzejewski@umcs.pl (K.J.)

**Keywords:** adaptation, biotransformation, UV/NTG mutagenesis, psychrotrophs, terpenes

## Abstract

Mutagenesis and adaptation of the psychrotrophic fungus *Chrysosporium pannorum* A-1 to the toxic substrate β-pinene were used to obtain a biocatalyst with increased resistance to this terpene and improved bioconversion properties. Mutants of the parental strain were induced with UV light and *N*-methyl-*N*′-nitro-*N*-nitrosoguanidine. Mutants resistant to β-pinene were isolated using agar plates with a linear gradient of substrate concentrations. Active mutants were selected based on their general metabolic activity (GMA) expressed as oxygen consumption rate. Compared to the parental strain, the most active mutant showed an enhanced biotransformation ability to convert β-pinene to *trans*-pinocarveol (315 mg per g of dry mycelium), a 4.3-fold greater biocatalytic activity, and a higher resistance to H_2_O_2_-induced oxidative stress. Biotransformation using adapted mutants yielded twice as much *trans*-pinocarveol as the reaction catalyzed by non-adapted mutants. The results indicate that mutagenesis and adaptation of *C. pannorum* A-1 is an effective method of enhancing β-bioconversion of terpenes.

## 1. Introduction

Terpenes are valuable natural substrates commonly used in the production of fine chemicals. Turpentine, obtained from biomass and also as a side product of the softwood industry, is rich in monoterpenes, such as α-pinene and β-pinene, which are widely used as raw materials in the synthesis of flavors, fragrances, and pharmaceutical compounds [1,2]. Oxidative transformation of abundant and low-priced monoterpenes holds considerable potential for the production of a wide variety of different terpenoid derivatives which are difficult and often costly to obtain directly from plant species. Oxygenated monoterpenes are the main flavor and fragrance impact molecules of essential oils. Many of them have beneficial effects on health [3,4,5], including anti-inflammatory activity [6]. However, their high volatility, poor solubility in aqueous media, and natural toxicity to microorganisms still limit the use of these compounds in applied biocatalysis. These limitations can be overcome by slow/gradual addition of terpene into the reaction medium, use of biphasic systems containing biocompatible organic solvents or a co-solvent, and in situ product removal techniques [7,8,9].

β-Pinene is commercially available at US$ 36 per kg, whereas the price of its oxygenated derivative, *trans*-pinocarveol, can be as high as US$ 134,000 per kg [10,11]. There are data regarding the antimicrobial properties of β-pinene, used either as a purified chemical or as a constituent of essential oils [12,13]. Some studies have also demonstrated that β-pinene and *trans*-pinocarveol, contained in the oils, show various biological effects [14,15,16,17,18].

Some microbial isolates have been found capable of transforming β-pinene into α-terpineol [19]. However, the rate and direction of the conversion are often inadequate for the process to be economically viable [20].

Strain developers have searched for improved strains among random survivors of mutagenesis. Even though the outcome of classical mutagenesis is difficult to predict, and the selection of mutants is always phenotypic, it still remains a fact that many producer strains with enhanced productivity currently used in industrial processes have been generated by random mutagenesis [21,22,23]. In contrast to genetic engineering methods, classical mutagenesis facilitates obtaining highly efficient mutants in an easy and rapid way without specialized knowledge about microbe genomes. To obtain the required mutants, experimenters have enriched cell populations by culturing them in special environmental conditions, toxic to most cell types but less toxic or non-toxic to a desired minority of cells [24,25,26,27,28]. Induced mutation has been successfully used in a number of microbial processes (other than biotransformation) employed in the production of useful end products [27,28,29].

Organic-solvent-tolerant mutants of bacteria can be selected using mutagen treatments or genetically engineered from solvent-sensitive parental strains [30,31,32]. Microbial tolerance to organic solvents can be improved by transforming cloned genes which encode various proteins involved in this tolerance, located in the cytoplasm or the inner or outer membrane [33,34]. Overexpression of genes which confer tolerance to specific organic solvents results in enhanced tolerances, which can be put to practical use [35].

Mutants resistant to a particular type of abiotic stress were better adapted to other stress conditions, as expressed by their enzymatic activities. The ability of one stress condition to provide protection against other stresses is referred to as cross-protection [36]. Adaptation processes are used in many branches of industrial biotechnology, including biotransformation of chemical compounds [37,38,39], biofuel production [40,41], and polymer synthesis [42].

In a previous paper, we demonstrated that the psychrotrophic fungus *Chrysosporium pannorum* A-1 showed promise for the biotransformation of (1S)-(−)-α-pinene because it could be used at 20°C. This is an advantage in bioprocess involving volatile terpenes. Thus far, it has also given the best yields of verbenol and verbenone (722 and 176 mg/L, respectively) among the microorganisms studied [43].

In the present experiments, mutagenesis (UV irradiation and *N*-methyl-*N*′-nitro-*N*-nitrosoguanidine (NTG)) and adaptation to a toxic substrate (β-pinene) were used to select mutants of the psychrotrophic fungus *C. pannorum* A-1 characterized by an improved efficiency of biotransformation of β-pinene to the main product *trans*-pinocarveol.

## 2. Results and Discussion

Biotransformation of hydrophobic terpenes is limited by their toxicity to microbial biocatalysts. The metabolic activity of microorganisms, which is strongly dependent on environmental parameters, may also be affected by stressful conditions. It has been shown that the effectiveness of biotransformation largely relies on the interactions between the biocatalyst and environmental stressors, which may increase the yields of biotransformation products [44]. The influence of preincubation of the fungus *C. pannorum* A-1 under different stress conditions (organic solvents, medium pH, and temperatures) on its activity in oxidative bioconversion of α-pinene to verbenone and verbenol was examined in our previous study [44]. Since many genes are responsible for resistance to abiotic stresses, classical mutagenesis methods combined with adaptation can provide an alternative for fast generation of efficient mutants. Mutants characterized by resistance to different stress factors can be of potential use for biotransformation of toxic organic compounds. Classical mutagenesis is especially important when the metabolic pathways and the genome in the host strain have not yet been determined, as is the case with *C. pannorum*, the species used in the present study.

The lethal effect of UV used along with 0.01% NTG was studied by exposing *C. pannorum* A-1 to these mutagens for different time periods. The results show that the survival rate dropped significantly at a UV exposure time longer than 10 min and an NTG exposure time longer than 5 min. More precisely, it was found that 10 min of exposure to UV and 5 min of exposure to 0.01% NTG resulted in an approximately 9.6% survival rate, while 10 min of UV irradiation combined with 10–15 min of exposure to 0.01% NTG gave a survival rate of 1.6%. When exposure times were prolonged to 20 min for 0.01% NTG and 15 min for UV, the survival rate decreased dramatically to 0.37% (Table S1).

The gradient plate technique was found to be applicable to our mutant selection procedure, which uses β-pinene, a compound poorly soluble in water. We confirmed the linear concentration gradient of this substrate in agar plate regions (by Gas Chromatography (GC) analysis, data not shown). The gradient was consistent with the number of colonies growing in particular agar plate regions (Figure S1). A total of 137 mutants that grew well in the regions with the highest β-pinene concentration were isolated, and their general metabolic activity (GMA) was determined. Thirty mutants characterized by a higher GMA compared to non-treated strain were selected for further experiments. They were transferred to adaptive medium containing β-pinene and to non-adaptive malt agar medium (Figure S2).

**Figure 1:**
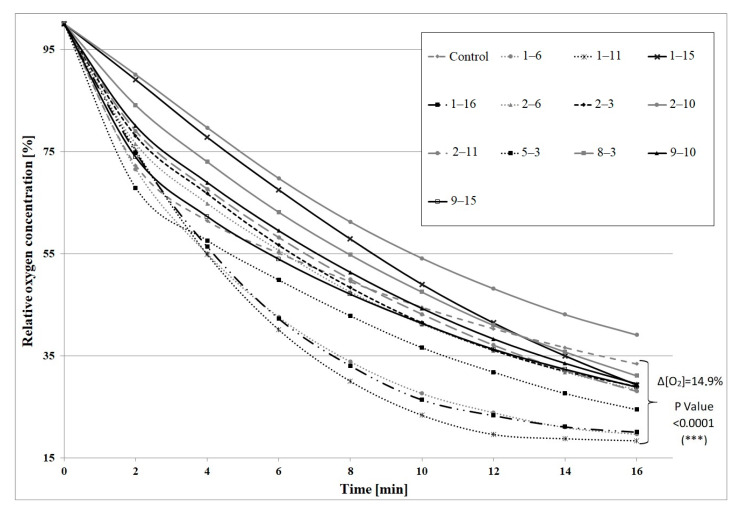
General metabolic activity (GMA) of 12 mutants of *C. pannorum* A-1 adapted to toxic β-pinene and selected for their high biotransformation ability. A non-treated wild strain was used as a control. Biomass concentration in each sample was 5 g dry mass per L.

**Figure 2:**
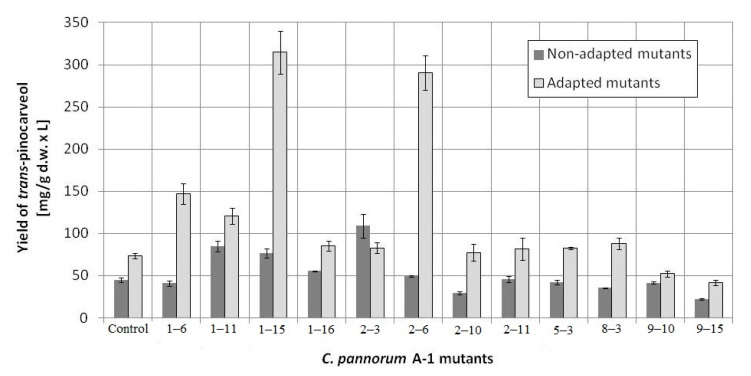
Effect of mutagenesis and adaptation to the substrate β-pinene on the biotransformation yield of 12 most active mutants of *C. pannorum* A-1. Non-treated parental strain and parental strain adapted to pinene were used as controls.

Then, a second selection round was performed on the basis of the mutants’ GMA. The results for the most active mutants are given in Figure 1 and oxygen uptake rates are shown in Table S2. In this selection round, twelve most active mutants were selected, which constituted 8.8% of all mutants. They were used for β-pinene biotransformation. Some of the mutants showed a substantially increased capacity to transform β-pinene to *trans*-pinocarveol (Figure 2). The most active non-adapted mutant (2–3) was characterized by a 2.4-fold increased biotransformation activity, whereas the average increase in activity among non-adapted mutants was 1.2-fold compared to the parental strain (*p* < 0.05) (ANOVA, Tukey`s test). The efficiency of the biotransformation performed using mutants adapted to 1% β-pinene was on average 1.7-fold higher (per 1 g of dry weight of mycelium) than for non-adapted mutants. Large increases in biotransformation activity were observed for mutants: 2–6 (4.0-fold), 1–6 (2.4-fold), and 1–11 (1.8-fold). The most active mutant (1–15) was characterized by a 4.3-fold and 7-fold higher biotransformation yield in comparison to the adapted control and the non-adapted control, respectively (*p* < 0.05) (ANOVA, Tukey`s test). The results are presented in Figure 2.

**Figure 3:**
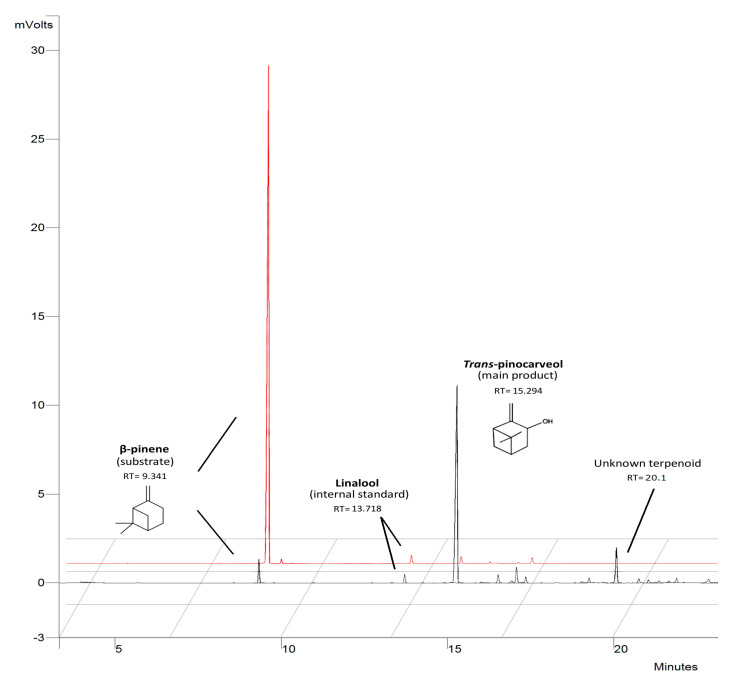
Gas Chromatography with Flame-Ionization Detection (GC-FID) chromatogram of main terpenoids obtained after 48 h of biotransformation of β-pinene by mutants of *C. pannorum* A-1. The initial substrate concentration was 1% (*v*/*v*). The red chromatogram refers to an abiotic control.

The main product of β-pinene biotransformation by the examined mutants was *trans*-pinocarveol, except for mutant 9–15, which accumulated substantial amounts of an unidentified product with a retention time of 20.1 min (the mass spectrum of this product is shown in Figure S3). This compound is probably produced by further metabolization of *trans*-pinocarveol, which might be catalyzed by a nonspecific enzyme induced during pinene biotransformation. This is more likely than not, as the fungal mutants neither grew on *trans*-pinocarveol as sole carbon source nor biotransformed it (data not shown). The fact that only one main product, with small total amounts (≤ 35%) of side-products (Figure 3), was produced is advantageous because usually biotransformation processes yield mixtures of different compounds which are then more difficult to separate.

For the twelve most active mutants, β-pinene biotransformation efficiency was associated with the survivability of conidia (in the range from 1.6 to 9.6%) after mutagenesis (Table 1). The highest biotransformation efficiencies were obtained by the mutants which showed 9.6% conidia survivability. An increase in the biotransformation of α-pinene to verbenol using *Aspergillus niger* and *Penicillium* spp. after treatment with UV irradiation, colchicine, or ethyl methanesulfonate (EMS) was reported by Agrawal et al. [45]. The best results were obtained when UV irradiation was used: the biotransformation efficiency increased 15- and 8-fold, respectively, reaching 58.5 mg/g d.w. × L (mg of the product per gram of dry weight of the mycelium per liter aqueous phase) for *A. niger* and 19.5 mg/g d.w. × L for *Penicillium* spp. By contrast, both strains produced substantially lower quantities of verbenone for all the treatments used, relative to the wild-type strain. In addition, compared to our study, those authors obtained much lower (5.4–16.2-fold) product concentrations [45].

Phenomena associated with the adaptation of microbial cells to high concentrations of toxic substrates have been investigated in various studies [46,47,48,49,50,51]. For example, exposure of yeast cells to a stepwise increase in the level of ethanol stress has been shown to be an effective method of obtaining ethanol-tolerant yeast strains [46]. Compared with the parental strain, chemically mutagenized and spontaneous mutants exhibited increased acclimation and elevated growth rates when cultivated in sublethal ethanol concentrations, and they showed an increased survivability in lethal ethanol concentrations. Their higher tolerance to ethanol was due to the fact that they showed elevated glycerol production rates, which, in turn, was associated with an increase in the ratio of oxidized and reduced forms of nicotinamide adenine dinucleotide (NAD^+^/NADH) in an ethanol-compromised cell, which stimulated the yeast cells’ glycolytic activity [52].

**Figure 4:**
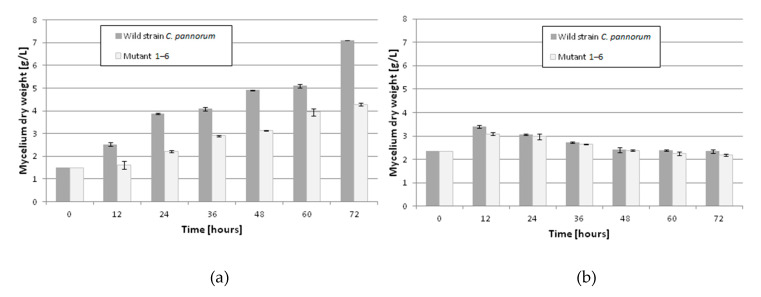
Cell growth rate of wild-type strain and mutant 1–6 of the fungus *C. pannorum* A-1 during biotransformation on rich basal medium (BM) (**a**) and 1% glucose medium containing phosphate buffer (**b**). Biotransformation conditions: temperature, 20 °C; initial β-pinene concentration, 1% (*v*/*v*). Bars represent the standard deviation of two independent samples.

**Figure 5:**
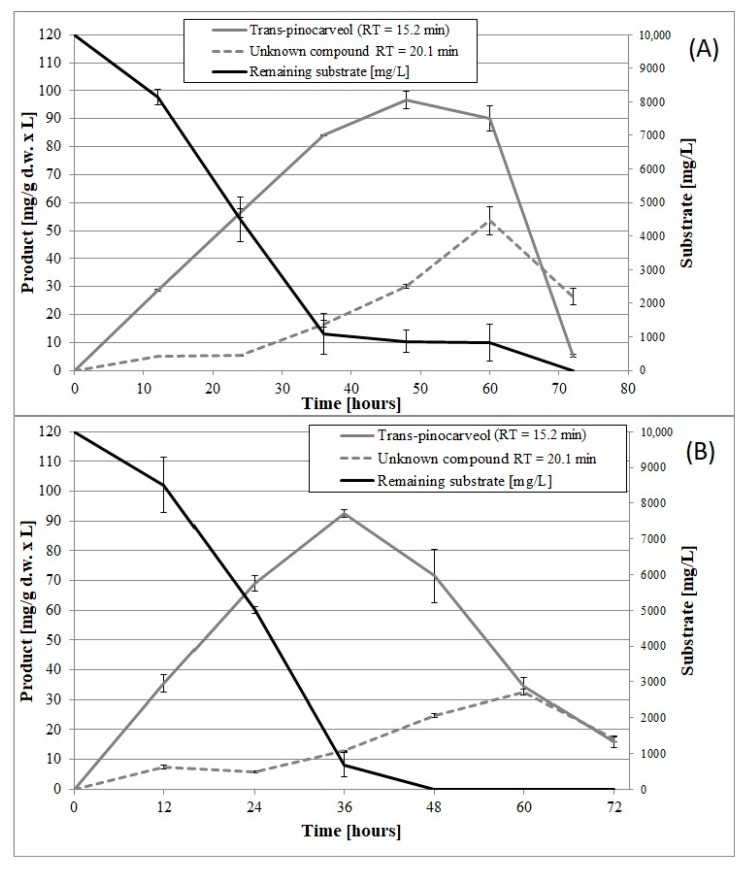
Time course of β-pinene biotransformation on basal medium (BM) by the wild-type strain (**A**) and mutant 1–6 (**B**) of the fungus *C. pannorum* A-1. An identical biomass concentration (1.5 g dry mass per L) was used in each sample. Bars represent the standard deviation of two independent samples.

Until now, no data have been published on the induction of psychrotrophic mutants using mutagenesis and adaptation to toxic substrates. There are also no reports on the ability of psychrotrophic fungi to biocatalyze the oxidation of β-pinene at temperatures below 25 °C. In this study, we attempted to fill the gap in previous research by establishing whether the mutants of the fungus *C. pannorum* A-1 had the biotechnological capability to catalyze an oxidation reaction at a low temperature and we also wanted to select mutants which produced high yields of high-value terpenoids from β-pinene. As far as we know, there are also no comprehensive reports regarding the effect of cell oxygen uptake on the biocatalytic activity of fungi. In our first experiments (Figure 1 and Figure 2), no correlation was found between oxygen consumption by the fungal strains and the efficiency of the β-pinene biotransformation reaction estimated after 48 h. Therefore, the kinetic experiment was performed to analyze differences in the biocatalytic activities between the parental strain and mutant 1–6, which differed in oxygen uptake rate (k = 0.0271 and k = 0.0445, respectively) (Table S2). Attention was paid to some of the most important biocatalyst characteristics, such as resistance to process conditions and a short biotransformation time. An increase in biomass growth was observed during biotransformation on basal medium (BM) (Figure 4A). A higher biomass yield was produced by the parental strain (*p* < 0.001) (ANOVA, Tukey`s test). On the rich BM medium, the metabolism of the parental strain was geared toward utilization of carbon, phosphorous and nitrogen sources (i.e., primary metabolism). In consequence, the higher biomass yield was accompanied by a higher *trans*-pinocarveol yield, compared to the mutant (Figure 5). However, in these conditions, the mutant adapted to β-pinene showed a shorter biotransformation time (36 h) in comparison to the parental strain (48–60 h) (*p* < 0.05) (ANOVA, Tukey`s test). In the case of the mutant, a lower yield of *trans*-pinocarveol was obtained along with a higher substrate depletion (Figure 5B). This indicates that further metabolization of the product must have occurred.

**Figure 6:**
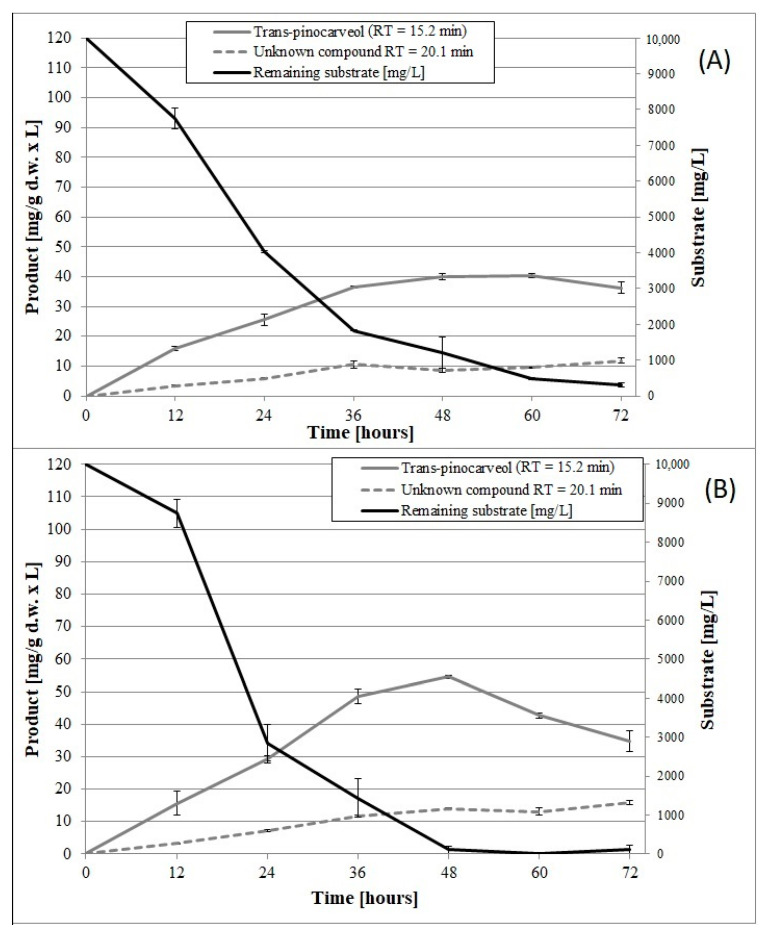
Time course of β-pinene biotransformation by the wild-type strain (**A**) and mutant 1–6 (**B**) of the fungus *C. pannorum* A-1 in phosphate buffer containing 1% glucose. An identical biomass concentration (2.35 g dry mass per L) was used in each sample. Bars represent the standard deviation of two independent tests.

When incubated in phosphate buffer, mutant 1–6 produced an about 1.4-fold higher yield of *trans*-pinocarveol from β-pinene compared to the parental strain (Figure 6) (*p* < 0.05) (ANOVA, Tukey`s test). These two strains also differed in the optimum biotransformation time (depending on the main product), which varied from 48 to 72 h for the parental strain, and was 48 h for the mutant. The amount of dry mycelium of both strains decreased gradually over a period of 60 h after biotransformation on the buffer medium (Figure 4B), due to autolysis induced by nutrient deprivation.

The fact that the mutant produced higher yields of the unknown product (RT = 20.1 min) and caused higher substrate depletion (48 h) compared to the parental strain (72 h), (p < 0.05) (ANOVA, Tukey`s test) confirmed the higher biocatalytic activity of the former. The results showed that biotransformation efficiency could be improved by using psychrotropic mutants characterized by higher oxygen consumption rates relative to the wild-type strain. Our results are consistent with the findings of Weber et al., who observed an appreciable increase in the rate of oxygen consumption by the mesophilic fungus *Cladosporium sphaerospermum* after addition of biodegradable substrates (i.e., benzyl alcohol, benzaldehyde, and catechol) [53].

A comparison of the biocatalytic efficiencies of the mutants adapted versus non-adapted to the substrate showed that the former produced higher yields of the biotransformation product (Figure 3). Cell adaptation to a solvent, substrate, and/or product has been found to be a successful strategy for reducing reagent inhibition in bioconversion systems [54,55]. Pre-delivery of low amounts of such compounds may result in increased synthesis of enzymes responsible for the transformation of xenobiotics [56]. Psychrotrophic fungi are adapted to survive in cold and very nutrient-poor environments, mainly due to the composition of their cell membranes and proteins. In addition, their enzymes are able to transform many xenobiotics into compounds that can be used as energy sources or ones that are less toxic to the cell [57,58,59]. The appearance of xenobiotics in the environment may cause stress to microorganisms, which, in turn, may induce adaptation mechanisms, such as changes in membrane composition or activation of genes responsible for the synthesis of chaperone proteins and catabolic enzymes, mainly cytochromes P450 [60,61]. Adaptation of microorganisms to a toxic substrate or induction with sublethal concentrations thereof often gives them an appropriate phospholipid profile [62,63]. In addition, bacterial cells (e.g., *Pseudomonas rhodesiae*) exposed to such harmful solvents as toluene and chloroform often undergo permeabilization, which increases biotransformation rates by improving the diffusion of products and substrates through the cell membrane [64]. Moreover, adaptation responses to one sub-lethal stress can lead to reduced susceptibility to a different stress (cross-protection or cross-adaptation) [65].

**Figure 7:**
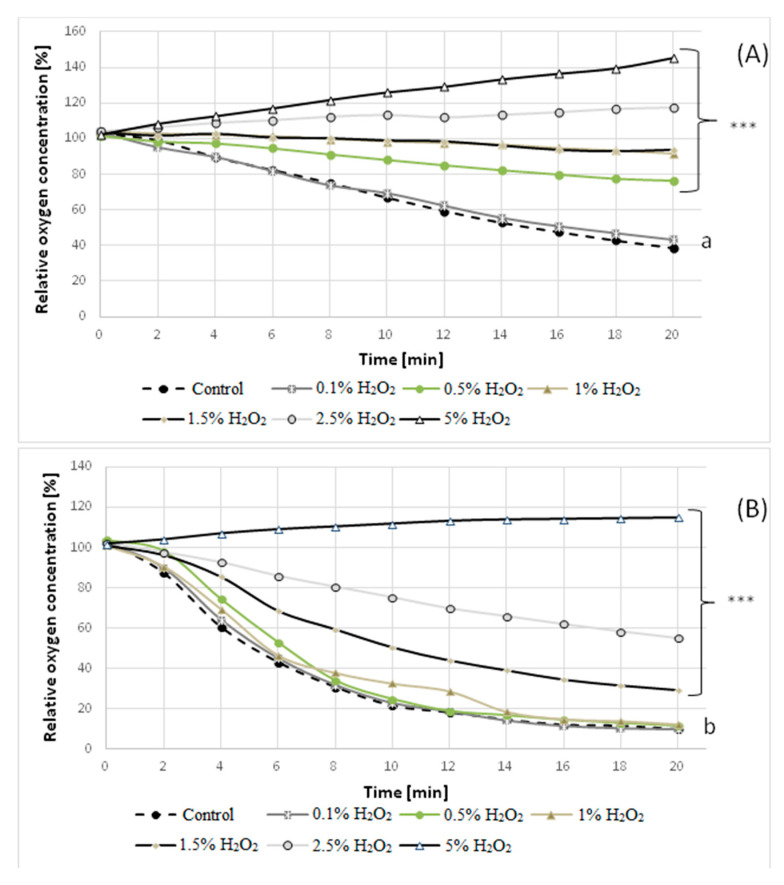
Oxygen consumption rate by the mycelium of the wild strain (**A**) and mutant 1–6 (**B**) of *C. pannorum* A-1 pre-incubated with different concentrations of H_2_O_2_. ***(*p* < 0.0001) indicate statistically significant differences with respect to the control (mycelium incubated without the addition of H_2_O_2_). Lowercase letters (a, b) indicate that the differences between control wild strain and the 1–6 mutant are significant (*p* ≤ 0.0001).

Since sublethal stresses in the β-pinene environment might induce stress-adaptive responses that could possibly make fungal cells resistant to other lethal stress factors [66], in the next experiment, we examined the resistance of *C. pannorum* A-1 strains to hydrogen peroxide. A remarkable increase in the decomposition of H_2_O_2_ was observed for mutant 1–6 relative to the wild strain. A 3-fold higher concentration of H_2_O_2_ (1.5% v/v) was required to inhibit the metabolic activity of the mutant compared to the wild strain (0.5% *v*/*v*), as shown by detection of H_2_O_2_ (undecomposed by catalase) in the medium after 1 h incubation (Table 2). This result was confirmed by measuring the GMA of the H_2_O_2_-stressed mycelia (pre-incubated with H_2_O_2_ at concentrations of 0.1–5% *v*/*v*). The examined concentrations of H_2_O_2_ above 0.1% caused a gradual fall in the rate of oxygen uptake by parental *C. pannorum* A-1 (Figure 7A). Incubation in the presence of 1.0% of H_2_O_2_ led to an almost total inhibition of oxygen consumption by the mycelium of this strain. A much smaller respiration-suppressing effect was observed for mutant 1–6, for which the total inhibitory concentration of H_2_O_2_ was as high as 5% (Figure 7B). This finding shows that biotransformation of volatile terpenes can be run under unconventional oxygenation of the culture with hydrogen peroxide (H_2_O_2_) as a supplemental oxygen source [67,68].

We showed that the mutants characterized by a higher GMA exhibited both a higher biotransformation rate and a higher resistance to H_2_O_2_-induced oxidative stress, compared to the wild strain; cell adaptation to monoterpene, β-pinene enhanced this effect. Sequential adaptation of *P. digitatum* NRRL 1202 to small doses of (R)-(+)-limonene led to a 12-fold increase in the efficiency of the biotransformation of this compound to α-terpineol in relation to an uninduced strain [69]. Adaptation of *Rhodococcus erythropolis* cells to increasing concentrations of carveol (substrate) and carvone (product) in n-dodecane prior to biotransformation resulted in an 8.3-fold increase in the carvone production rate [70]. Induction with styrene of *Pseudomonas* sp. VLB120DC, a strain capable of processing styrene into pure enantiomeric (S)-styrene oxide, allowed to obtain a specific oxygenase activity of 60–70 U/g cell dry weight (CDW), where the activity of uninduced cells remained at the level of 0.7 U/g CDW [42]. *Salmonella enterica* serovar Enteritidis adapted to ethanol developed cross-tolerance to malic acid, resulting in a doubling of the minimum bactericidal concentration and increased cell survival rates [71].

## 3. Materials and Methods

### 3.1. Chemicals

(1S)-(−)-β-Pinene (98%), N-methyl-N′-nitro-N-nitrosoguanidine (NTG) (97%), and *trans*-pinocarveol (≥96%) were purchased from Sigma-Aldrich, St Louis, MO, USA. (−)-Linalool, at a purity of >97%, was obtained from Fluka, Buchs, Switzerland, and was stored at 4 °C. Hydrogen peroxide (30%) and potassium iodide (>99%) were obtained from POCH, Gliwice, Poland.

### 3.2. Fungus and Media

The experiments were performed using the psychrotrophic fungus *C. pannorum* A-1 as the host strain. Fungal cells had been isolated from soil samples collected in the Arctic tundra (West Spitsbergen) [72]. Prior to the experiments, the cells had been maintained on malt agar slants at 4 °C, and subcultured every month. The cultivation and bioconversion procedures were carried out using liquid basal medium (BM) composed of glucose 1%, malt extract 1%, peptone 0.5%, and yeast extract 0.5% (pH = 6.14). Adaptation experiments were carried out in adaptive medium M1 composed of agarized BM diluted 10-fold with distilled water with an addition of β-pinene at a concentration of 1% (*v*/*v*). β-pinene-resistant mutants were selected and isolated on the following solid media: 1. a gradient medium consisting of BM and water with 1% β-pinene (M2); 2. a minimal medium composed of water, mineral salts [K_2_HPO_4_ 0.1%, (NH_2_)_2_SO_4_ 0.5%, MgSO_4_ 0.02%], and 1% β-pinene (M3); β-pinene was dispersed as an emulsion in 10-fold diluted BM using 0.01% Tween 80. Agarized BM was used to determine conidial survival.

### 3.3. Induction, Adaptation, and Selection of Mutants

Mutants were induced by treating a conidial suspension (2 × 10^6^ spores mL^−1^) with UV irradiation for 10 or 15 min (960 erg/mm^2^) and NTG at a final concentration of 0.01% for 5 to 20 min (UV-NTG method) [73] with magnetic stirring (150 rpm) under room temperature according to the program given in Table S1. After mutagenization, the surviving spores were spread on dishes with agarized BM and incubated 4 days at 20 °C. The number of colonies formed was compared to control plates with non-treated spores. At this stage, the drop in viability was over 90%. For further experiments, random mutants which grew well in the presence of β-pinene were isolated. For this purpose, the colonies were passaged several times on solid BM with the addition of 1% β-pinene and then sown on M2 medium with a linear gradient of β-pinene concentrations according to the Gradient Plate technique [74].

To confirm the linear concentration of β-pinene in the agar plates, pieces of M2 medium were extracted and analyzed using GC. Agar discs (6 × 8 in diameter) were cut with a sterile cork borer from at least five regions. They were then extracted with EtOAc and subjected to pinene quantification.

For initial adaptation, a fragment of the mycelium of each mutant was transferred onto a Petri dish containing 10-fold diluted BM (M1) with a hollow in the center filled with β-pinene (50 μL). Colonies which grew the best were picked, and their general metabolic activity (GMA) was determined as the oxygen consumption rate in phosphate buffer according to the procedure described below. The most metabolically active mutants were transferred onto adaptive medium M1 containing β-pinene (adapted mutants) or onto wort medium M1 without the substrate (non-adapted mutants). The mutants were incubated for a month, after which another selection round was carried out based on GMA.

Mutants characterized by the highest GMA were transplanted onto slants with adaptive medium M1, stored at 4 °C, and subcultured every month.

### 3.4. General Metabolic Activity (GMA) Assay

The general metabolic activity of the mycelium was determined as the oxygen uptake rate using a calibrated VisiFerm DO Arc 225 electrode (Hamilton Robotics, Bonaduz, GR, Switzerland). Shaking flask cultures (150 rpm) of the mutants were grown in liquid BM at 20 °C. After two days of growth, 25 mL mycelial culture of *C. pannorum* A-1 containing a fixed amount of fungal mycelium (5 g of dry weight per L) was aseptically transferred into 100 mL beakers containing 50 mL of 0.05 M McIlvaine buffer (pH 6) with an addition of 0.5% glucose. Finally, the samples were aerated to a dissolved oxygen (DO) concentration of 100% air saturation and incubated with magnetic stirring (150 rpm) under a controlled temperature (20 °C). The electrode was calibrated before each experiment by measuring its signal in the air-saturated McIlvaine buffer prior to the addition of the mycelium. The values of the readings were expressed as percent of dissolved oxygen saturation and were then converted to oxygen uptake rate k [% × s^−1^]. In all experiments, mycelium was sampled directly from the pellet culture. Care was taken to obtain samples representative of the different pellet sizes and mycelium weights. Three independent experiments were performed. The results are reported as means of all replicates.

### 3.5. Operation at Shake Flask Scale

To perform biotransformation experiments, 25 mL of sterile basal medium (in 100-mL Erlenmeyer flasks) was uniformly inoculated with 1.5 mL of *C. pannorum* spore suspension (at concentration of 2 × 10^6^ mL^−1^) and cultivated at 20 °C on a rotary shaker (150 rpm). The fungal spore suspension was prepared as an inoculum using 6-day old spores pre-grown at 20 °C on agar slants, which were then harvested, filtered through glass wool to remove hyphal fragments, and washed twice with sterile distilled water with 0.01% Tween 80, pH 5.0. Biotransformation was started by directly adding 1% *v*/*v* of β-pinene to the pre-grown 2 day-old mycelial culture using the procedure described in [43]. All β-pinene biotransformations were carried out in parallel with controls, in identical conditions, using heat-inactivated microorganisms which had been autoclaved for 15 min at 121 °C.

For the kinetic experiment, mycelia of the parental strain and mutant 1-6 previously cultured for 2 d using the procedure described above, were harvested washed twice with sterile 0.1 M McIlvaine buffer, pH 5.0. Next, equal amounts (by weight) of biomass were aseptically transferred to Erlenmeyer flasks containing 25 mL of sterile media. Biotransformation of β-pinene (1% (*v*/*v*)) was performed on BM and on phosphate buffer, pH 6, containing 1% glucose, using 1.5 and 2.35 g of dry mass per L, respectively. The entire contents of three flasks were sacrificed each time at regular intervals (0, 12, 24, 36, 48, 60, 72, and 96 h) to assay the products of biotransformation. Growth was followed by measuring the dry weight of mycelium. Mycelial biomass was determined gravimetrically, after washing with 0.1 M phosphate buffer, by drying to a constant weight in an oven at 105 °C for 3 h. It was expressed as g/L dry weight.

The biotransformation yield was defined as the quantity of the product per gram of dry weight of the mycelium per liter aqueous phase (mg/g d.w. × L).

### 3.6. Effect of Hydrogen Peroxide Concentration on the Metabolic Activity of the Parental and the Mutant Strain of C. pannorum A-1

Identical amounts (2.65 g of dry weight per L) of two-day-old mycelia of the parental strain and mutant 1-6 were washed twice with sterile 0.1 M phosphate buffer and transferred to 100 mL Erlenmeyer flasks containing 40 mL dissolved BM with an addition of hydrogen peroxide at concentrations of 0.1, 0.5, 1.0, 1.5, 2.5, and 5% (*v*/*v*). After 60-min incubation at 20 °C with magnetic stirring (150 rpm), the residual content of H_2_O_2_ was estimated in 1 mL aliquots of the medium using potassium iodide and starch solution as an indicator. The pre-incubated biomasses were filtrated, washed twice with sterile 0.9% NaCl solution, and then fixed amounts of the mycelia were suspended uniformly in 0.05 M McIlvaine buffer (pH 6) with an addition of 1% glucose in order to measure the GMA of living mycelia, according to procedure described in the ‘General metabolic activity assay’.

### 3.7. Biotransformation Analysis

After the specified biotransformation time, the biomass was harvested by filtration, and the liquid for product recovery was extracted twice with an equal volume of diethyl ether in a separation funnel for 10 min. Before extraction, 250 μL of a 0.1% internal standards (IS) solution in hexane was added to the filtrate. The ether fraction was collected and concentrated on rotary vacuum evaporators (BUCHI, Rotavapor R-200/205, Flawil, Schwitzerland) at a water bath temperature of 40 °C under mild pressure of 800 mbar. The residues obtained were dissolved in 6 mL of hexane. Gas chromatography with flame-ionization detector (GC-FID) (VARIAN, Palo Alto, California, USA) and mass specta coupled to gas chromatography (GC-MS) (Thermo Finnigan, Trace DSQ GC/MS Ultra Chromatograph, Austin, TX, USA) analyses of terpenes were conducted using the method reported previously [43]. Peaks were identified by fitting the mass spectra to those from standard library database systems (HP Wiley, NIST) and the MassFinder library and by comparing the GC retention indices to those of standard compounds. Biotransformations were performed in three replicate samples, and the analyses were carried out in duplicate. The error associated with the GC quantification of the samples was ±6% and was quoted for a confidence interval of 94%. The data are reported as mean values.

### 3.8. Statistical Analysis

The experiments were carried out in triplicate. The results shown in Figure 1, Figure 4, Figure 5 and Figure 6 were analyzed by a one-way analysis of variance (one-way ANOVA) followed by Tukey`s post-hoc test for multiple comparisons. Data from the comparison of the control strain and the 1–6 mutant were analyzed statistically using the unpaired Student’s t-test (Figure 7). The statistical analysis was carried out using GraphPad Prism for Windows version 5.03 (GraphPad Software, San Diego, CA, USA). All results are presented as mean +/- SEM. *p* < 0.05 was considered statistically significant with a confidence level of 95%.

## 4. Conclusions

The present results demonstrate that the adapted mutants of *C. pannorum* A-1 are characterized by an enhanced resistance to stressful conditions. So far, however, they have shown moderate biocatalytic activities, giving relatively high *trans*-pinocarveol yields, which means they are still subject to optimization. The maximum *trans*-pinocarveol concentrations obtained in the experiment reported in the present paper were in the range of 147.2–314.7 mg/g d.w. × L. In our non-optimized flask system, we were able to biotransform of β-pinene worth US$ 0.36 to *trans*-pinocarveol with a value of US$ 67 (per 1 L of batch culture), using the most active mutant. This result indicates that *C. pannorum* A-1 and its mutants are efficient biocatalysts for the conversion of β-pinene to this valuable product. Further improvement in β-pinene biotransformation may be achieved by selecting blocked mutants of the isolated fungal strains, which would prevent further metabolization of the main product (*trans*-pinocarveol). Filtration-enrichment methods for selecting auxotrophs may be used for this purpose [75]. Moreover, optimization of the culture conditions in bioreactors is necessary for the strain to be utilized in larger scale bioconversion processes, as some parameters, such as oxygen supply or pH, cannot be controlled in a flask culture.

The results show that it is possible to increase the rate of positive mutations by using media with a gradient of the toxic substrate and performing rapid initial evaluation of the GMA of the examined mutants of *C. pannorum* A-1. The use of the gradient plate method and subsequent determination of the GMA of the isolated mutants greatly simplifies the selection of substrate-resistant strains. Direct screening has the advantage of significantly reducing the number of cultures isolated from plates, which would normally require productivity to be tested in shake flask cultures. This method may considerably improve the selection of β-pinene biotransformation-active microbial strains and also other cultures showing biotransformation activity toward toxic substrates.

## Figures and Tables

**Table 1:** Relationship between survivability of conidia obtained using mutagenesis and the efficiency of biotransformation of β-pinene to the main product *trans*-pinocarveol by mutants of *C. pannorum* A-1.

Variant of Mutagenesis	Survivability [%]	The Most Biotransformation-Active Mutants	Efficiency of Biotransformation [mg/g d.w. × L]
10 min UV + 5 min NTG	9.6	1–15 ^1^	314.7 (±25.3)
		1–6 ^1^	147.2 (±11.9)
		1–11 ^1^	121.4 (±9.5)
		8–3 ^1^	88.5 (±6.3)
		1–16 ^1^	85.6 (±6.0)
10 min UV + 10 min NTG	1.6	2–6 ^1^	290.8 (±20.4)
		2–3 ^2^	109.4 (±14.2)
		2–11 ^1^	82.2 (±13.1)
		2–10 ^1^	77.8 (±10.1)
		9–10 ^1^	52.6 (±3.7)

^1^ adapted mutants; ^2^ non-adapted mutant. NTG – *N*-methyl-*N*′-nitro-*N*-nitrosoguanidine.

**Table 2:** Comparison of resistance of the parental strain and mutant 1–6 of the fungus *C. pannorum* A-1 to hydrogen peroxide as measured by their H_2_O_2_ decomposition capacity.

Presence of Hydrogen Peroxide in the Medium After 1-h Incubation		
H_2_O_2_ Concentration % (*v*/*v*)	Wild-Type Strain	Mutant 1–6
Control	−	−
0.1	−	−
0.5	+	−
1.0	+	−
1.5	+	+
2.5	+	+
5.0	+	+

Control – mycelium incubated without an addition of H_2_O_2_; (*–*) – not detected; (+) – detected.

## Supplementary Materials

The following are available online, Figure S1: Agar plate with linear gradient of β-pinene concentration, Figure S2: Flowchart of the procedure for improving *C. pannorum* A-1 as a biocatalyst for β-pinene biotransformation, Figure S3: Mass spectra of unknown compounds; RI = 1275, RT = 20.1 min, Table S1: Variants of mutagenesis of the psychrotrophic fungus *C. pannorum* A-1 and their impact on survivability of conidia. Survivability was expressed as the number of colonies formed compared to control plates with non-treated spores after 2 days of incubation at 20 °C on agarized BM, Table S2: Oxygen uptake rate *k* [% × s^−1^] for the 12 most active GMA mutants and parental strain. The standard deviation was approximately 3%
