# Supplementary material for: Mutagenesis and Adaptation of the Psychrotrophic Fungus Chrysosporium pannorum A-1 as a Method for Improving β-pinene Bioconversion
Source: Molecules. 2020 Jun 2;25(11):2589. doi: 10.3390/molecules25112589 (PMC7321369; doi:10.3390/molecules25112589)
Supplement: Supplementary file 1 [file molecules-25-02589-s001.pdf]

molecules

MDPI

Article

Mutagenesis and Adaptation of the Psychrotrophic Fungus *Chrysosporium pannorum* A-1 as a Method for Improving β-pinene Bioconversion

Mateusz Kutyła, Jan Fiedurek, Anna Gromada, Krzysztof Jędrzejewski and Mariusz Trytek*

Department of Industrial and Environmental Microbiology, Faculty of Biology and Biotechnology, Maria Curie-Skłodowska University, Akademicka 19, 20-033 Lublin, Poland; mateusz.kutyla@umcs.pl (M.K.); janek@umcs.pl (J.F.); anna.gromada@umcs.pl (A.G.); krzysztof.jedrzejewski@umcs.pl (K.J.)
*Correspondence: e-mail1: mariusz.trytek@umcs.pl; e-mail2: mtrytek1@o2.pl; Tel.: +48-81-537-5958

Academid Editor: Josefina Aleu

Supplementary Material

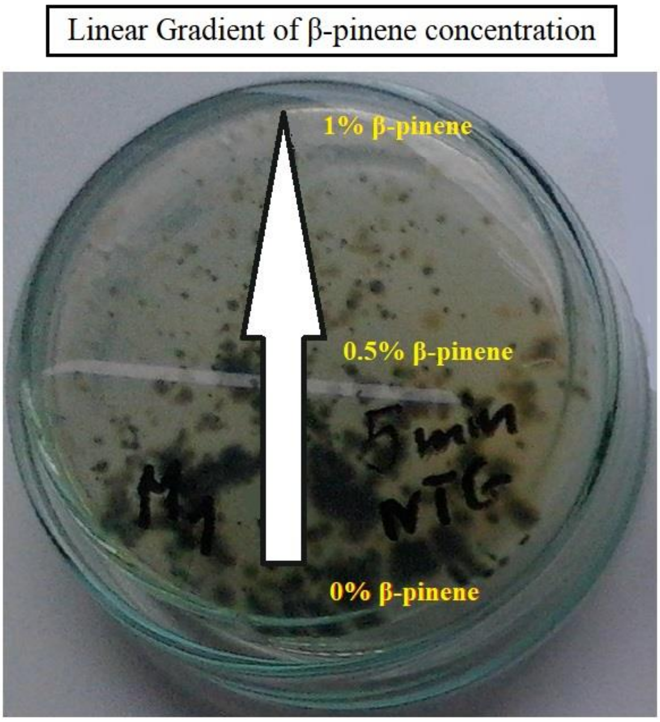

Figure S1. Agar plate with linear gradient of β-pinene concentration.

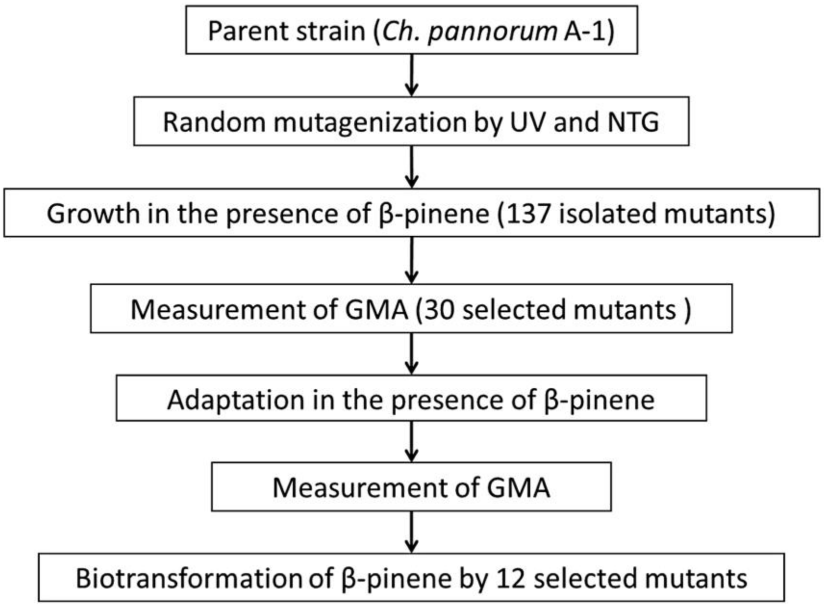

Figure S2. Flowchart of the procedure for improving *C. pannorum* A-1 as a biocatalyst for β-pinene biotransformation.

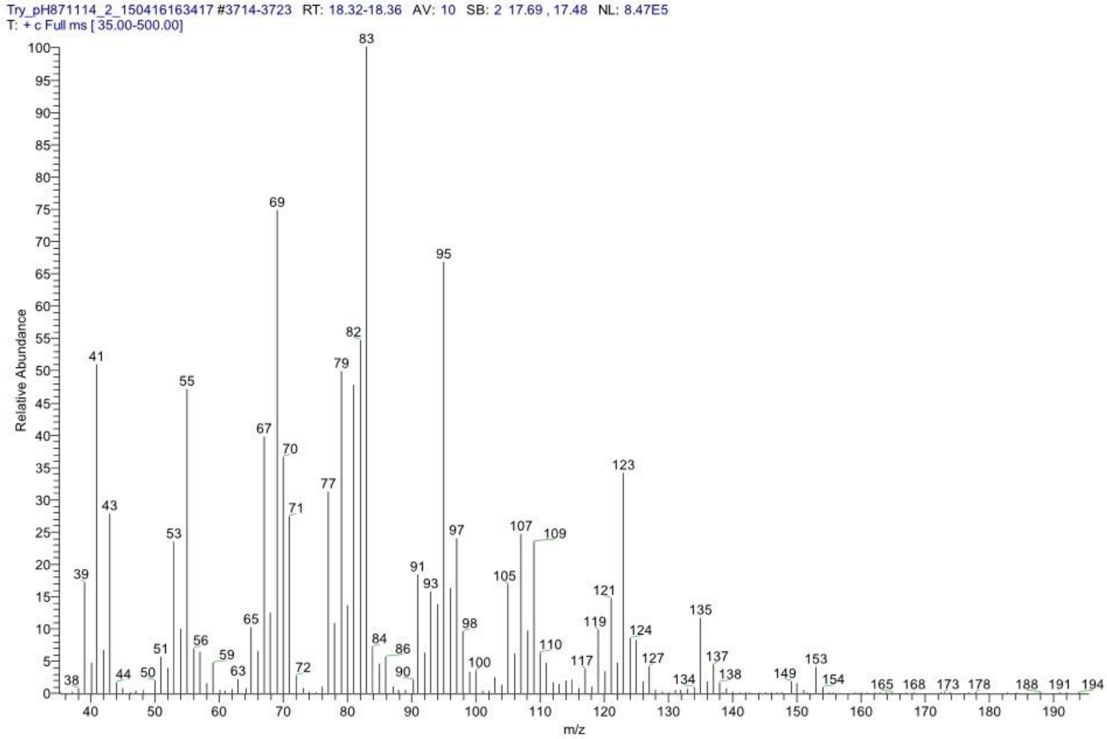

Figure S3. Mass spectra of unknown compounds; RI = 1275, RT = 20.1 min.

Table S1. Variants of mutagenesis of the psychrotrophic fungus C. pannorum A-1 and their impact on survivability of conidia. Survivability was expressed as the number of colonies formed compared to control plates with non-treated spores after 2 days of incubation at 20 °C on agarized BM.

Variants of Treatment with Mutagens	Survivability of Treated Conidia (%) (SD)
10 min UV + 5 min 0.01% NTG	9.6 (± 0.59)
10 min UV + 10 min 0.01% NTG	1.6 (± 0.09)
10 min UV + 15 min 0.01% NTG	1.6 (± 0.1)
15 min UV + 15 min 0.01% NTG	0.47 (± 0.03)
15 min UV + 20 min 0.01% NTG	0.37 (± 0.02)

Table S2. Oxygen uptake rate k [% × s−1] for the 12 most active GMA mutants and parental strain. The standard deviation was approximately 3%.

Strain Tested	Oxigen Uptake Rate k [% × s−1]
Parental	0.0271
1–6	0.0445
1–11	0.0490
1–15	0.0336
1–16	0.0451
2–6	0.0331
2–3	0.0331
2–10	0.0262
2–11	0.0336
5–3	0.0356
8–3	0.0314
9–10	0.0324
9–15	0.0318
